# Van der Waals MoS_2_/VO_2_ heterostructure junction with tunable rectifier behavior and efficient photoresponse

**DOI:** 10.1038/s41598-017-12950-y

**Published:** 2017-10-27

**Authors:** Nicoló Oliva, Emanuele Andrea Casu, Chen Yan, Anna Krammer, Teodor Rosca, Arnaud Magrez, Igor Stolichnov, Andreas Schueler, Olivier J. F. Martin, Adrian Mihai Ionescu

**Affiliations:** 1 Nanoelectronic Devices Laboratory (NanoLab), École Polytechnique Fédérale de Lausanne (EPFL), 1015 Lausanne, Switzerland; 2 Nanophotonics and Metrology Laboratory (NAM), École Polytechnique Fédérale de Lausanne (EPFL), 1015 Lausanne, Switzerland; 3 Solar Energy and Building Physics Laboratory (LESO-PB), École Polytechnique Fédérale de Lausanne (EPFL), 1015 Lausanne, Switzerland; 4 Istitut de Physique (IPHYS), École Polytechnique Fédérale de Lausanne (EPFL), 1015 Lausanne, Switzerland

## Abstract

Junctions between n-type semiconductors of different electron affinity show rectification if the junction is abrupt enough. With the advent of 2D materials, we are able to realize thin van der Waals (vdW) heterostructures based on a large diversity of materials. In parallel, strongly correlated functional oxides have emerged, having the ability to show reversible insulator-to-metal (IMT) phase transition by collapsing their electronic bandgap under a certain external stimulus. Here, we report for the first time the electronic and optoelectronic characterization of ultra-thin n-n heterojunctions fabricated using deterministic assembly of multilayer molybdenum disulphide (MoS_2_) on a phase transition material, vanadium dioxide (VO_2_). The vdW MoS_2_/VO_2_ heterojunction combines the excellent blocking capability of an n-n junction with a high conductivity in on-state, and it can be turned into a Schottky rectifier at high applied voltage or at temperatures higher than 68 °C, exploiting the metal state of VO_2_. We report tunable diode-like current rectification with a good diode ideality factor of 1.75 and excellent conductance swing of 120 mV/dec. Finally, we demonstrate unique tunable photosensitivity and excellent junction photoresponse in the 500/650 nm wavelength range.

## Introduction

VO_2_ is a strongly correlated functional oxide exhibiting an IMT at 340 K^1–4^. In the so-called insulating state at room temperature, VO_2_ is a semiconductor with a 0.6 eV band gap and monoclinic crystal structure, while above the transition temperature it exhibits a rutile structure and a metallic behavior with a resistivity drop up to 5 orders of magnitude in the bulk material^1,2^. The IMT can be induced by thermal, electrical, magnetic or optical excitations^3^ and is typically hysteretic. The electrically induced phase transition results in an extremely steep drop of the resistivity that has been exploited to realize VO_2_ two-terminal switches^3,4^. Coupled VO_2_ oscillators have been recently demonstrated together with their potential for neuromorphic pattern recognition applications^5^. The IMT of VO_2_ resistors connected in series to the source or gate of field-effect devices has been exploited to demonstrate switching slopes well below the 60 mV/dec Boltzman limit constraining the MOSFET subthreshold slope^6,7^. VO_2_ resistance-to-capacitance reversible reconfigurability is also appealing for RF applications^4^. However, the small band gap of VO_2_ in its insulating state determines a small resistivity and high leakage currents in VO_2_ switches. Moreover, the electrical induced phase transition requires typically several hundreds of µW to few mW of power^3,4^. VO_2_ three terminal gated devices have been proposed and investigated with the purpose of decreasing the voltage and current required to trigger the IMT, but the large electron density necessary for the phase transition at room temperature is difficult to achieve by exploiting the gate control only^8,9^. Large insulating state leakage and reduced gate control on the carrier density are currently two fundamental challenges on the road for VO_2_ based devices and circuits.

Here, we demonstrate for the first time a two terminal device based on a vdW heterostructure junction formed by VO_2_ and multilayer MoS_2_ flakes. Bulk-like MoS_2_ is an indirect band-gap semiconductor with a 1.3 eV band gap^10,11^, which forms a type II n-n heterojunction when in contact with VO_2_. The proposed device shows an excellent tunable rectification behavior and can be turned into a Schottky rectifier at high applied voltage or increased temperature by triggering the VO_2_ IMT. With respect to pure VO_2_ switches, this heterostructure delivers a lower leakage current in the diode subthreshold region and it could lead to the realization of more energy efficient VO_2_ devices. Compared to monolayer MoS_2_, multilayer flakes reduce the risk of damages due to the large power flowing in the devices when electrically actuated and provide lower contact resistance^12^. The fabricated devices proved to be photosensitive in the visible spectral range as a result of light absorption in the MoS_2_ side of the junction. We report here a complete photoresponse characterization including the impact of the thermally induced IMT of VO_2_. The extracted responsivities exceed the results reported for other photodiodes based on multilayer MoS_2_ flakes^11,13,14^.

## Results

### Electrical characterization of the heterojunction

**Figure 1:**
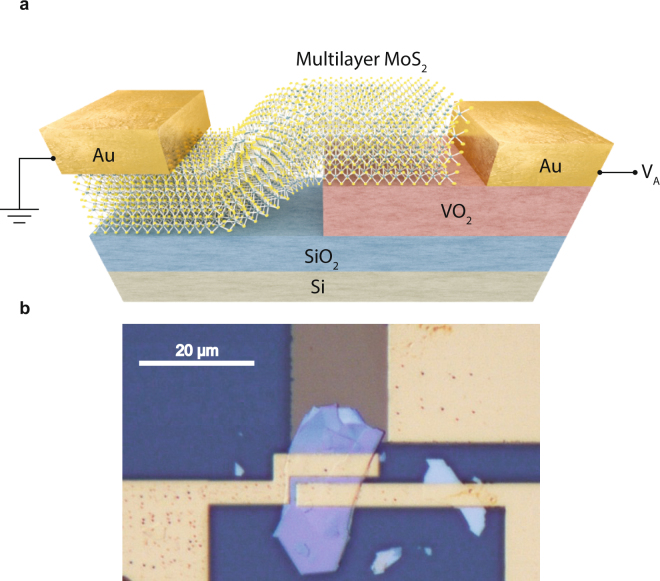
VdW MoS_2_/VO_2_ heterojunction schematic. (**a**) Three-dimensional schematic view of the MoS_2_/VO_2_ heterojunction with the bias configuration used in the experiments. The MoS_2_ contact is grounded while the bias is applied and swept on the metal contact on VO_2_. (**b**), Optical image of the fabricated heterojunction composed of a multilayer MoS_2_ flake and VO_2_ pre-patterned structure. Gold contacts to the two sides of the junction have been deposited to characterize the device. Two distinct set of contacts were deposited on the MoS_2_ flakes to verify their electrical behavior.

The schematic of the conceptual vdW MoS_2_/VO_2_ heterojunction together with the basic biasing scheme used for the experiments are depicted in Fig. 1a: the MoS_2_ contact is grounded and the voltage is applied and swept on the metal contact on VO_2_. An optical microscopy image of a fabricated MoS_2_/VO_2_ heterojunction with the electrical contacts to the two sides of the junction is reported in Fig. 1b. Two separate set of gold contacts were deposited on the MoS_2_ flake to verify their ohmic behavior (see Supplementary Information Fig. S1a). The thickness of the exfoliated flakes has been estimated by atomic force microscopy, and the devices described in the following have been realized with flakes thicknesses ranging from 80 to 100 nm. The VO_2_ and the SiO_2_ films are respectively 75 nm and 2 µm thick. Details on device fabrication are reported in the methods section. The X-ray diffraction study and resistivity curve of as deposited VO_2_ film, which demonstrates the absence of other vanadium oxide phases potentially detrimental for the device operation, are shown in Supplementary Information Figs S2a and S2b.

**Figure 2:**
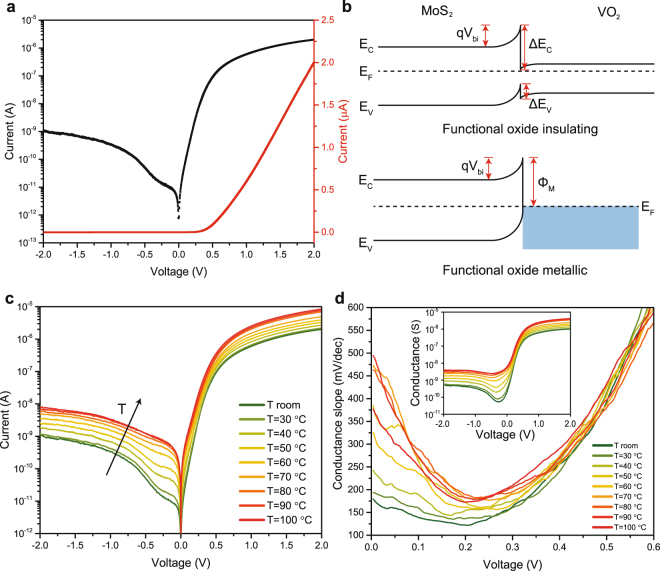
Electrical characteristic of the MoS_2_/VO_2_ heterojunction. (**a**) Electrical I–V double-sweep characteristic of device D1 at room temperature in semi-logarithmic (black) and linear (red) scales. The device presents a rectification behavior with no significant hysteresis. (**b**) Qualitative band diagram of the MoS_2_/VO_2_ heterojunction for the insulating and metallic phase of the functional oxide. The VdW gap at the junction is not represented and the polycrystalline nature of VO_2_ is not considered. (**c**) Evolution of the electrical characteristic of D1 with increasing temperature. Both forward and reverse current are boosted by the temperature increase, with a more pronounced enhancement in the window around the IMT temperature (60–80 °C). (**d**) Extracted conductance slope from the exponential regime of the forward current. The conductance slope increases across the VO_2_ IMT. Inset: conductance of device D1.

The current voltage characteristic of a first device (D1) at room temperature is shown in Fig. 2a. The MoS_2_/VO_2_ heterojunction exhibits a clear rectifying behavior with a rectification ratio I_F_/I_R_ larger than 10^3^ and an extracted I_F_/I_S_ exceeding 10^5^, where I_F_, I_R_ and I_S_ are respectively forward, reverse and saturation current. Under positive bias, the current shows an exponential increase that becomes linear under larger voltages because of the series resistance. The behavior of the junction can be explained in terms of the qualitative band diagram in Fig. 2b (see Supplementary Information Fig. S3 for more information). Both MoS_2_ and the insulating phase of VO_2_ are intrinsically n-type materials^15,16^. Since MoS_2_ workfunction is smaller than VO_2_ one, at the formation of the heterostructure electrons are transferred from the first to the second, resulting in the depicted band alignment, Fig. 2b. The large conduction band discontinuity (ΔE_C_), estimated by the affinity rule to be roughly 1 eV, is the main responsible for the asymmetric conduction of the junction. Applying a positive bias to VO_2_, electron injection from MoS_2_ conduction band to VO_2_ conduction band is favored, resulting in an exponential increase of the current. Conversely, under reverse bias electrons are injected from VO_2_ to MoS_2_, but they face the large ΔE_C_ barrier. Overall, the realized junction behaves as an n-n heterostructure with type II band alignment and is able to provide a good rectification. The device I–V characteristic in the exponential region can be approximated with the Shockley diode equation providing an extracted ideality factor of 1.75, while the non-saturating reverse current is better described by the Fang and Howard model^17^ specifically developed for n-n junctions and capable of analytically capturing the increase of diode current with the absolute value of the reverse bias (see Supplementary Information Fig. S4b,c).

The characterization of the heterojunction device in a wide range of increasing temperature is of particular interest because VO_2_ transition from the semiconductor to the metallic phase around 68 °C is expected to increase the conductance of the junction by collapsing the VO_2_ bandgap. The I–V curves measured with the temperature as a parameter are shown in Fig. 2c. Both forward and reverse current increase with temperature and a significant current enhancement is observed close to the IMT temperature. Above it, the temperature dependence of the characteristic is less pronounced and the device behaves as a Schottky rectifier. The band diagram of the junction with metallic VO_2_ is drawn in Fig. 2b. The Schottky barrier (Φ_M_) is estimated to be 0.3 eV larger than ΔE_C_, and the built-in voltage should increase slightly since VO_2_ work function is reported to increase across the IMT^2^. The reverse current increase with raising temperature can be explained with the growth of electron density in VO_2_ conduction band, changing from 10^18^ cm^−3^ in the insulating phase to 10^23^ cm^−3^ in the metallic one^18^. The boost of forward current can be attributed to the stronger thermionic injection in VO_2_ conduction band from MoS_2_ conduction band, but it is mitigated by the slight increase of the built-in voltage. The rectification ratio with metallic VO_2_ is slightly degraded with respect to the semiconductor junction but it remains larger than 10^3^. In order to characterize the junction conduction we extracted the conductance slope^19^ ${S}_{G}={({\rm{\partial }}logG/{\rm{\partial }}V)}^{-1}$ for the forward current at different temperatures (Fig. 2d). At room temperature, the device exhibits an excellent minimum *S*_*G*_ value of 120 mV/dec, which suggests that the heterojunction is abrupt and has a reduced density of interface defects. This observation is further supported by the absence of any hysteresis of the I–V characteristics (see Fig. 2a) in double-sweep measurements and by a low extracted quality factor of 1.75 in forward bias. The conductance slope increases across the MIT and tend to decrease after the phase transition.

### Demonstration of electrically induced reversible IMT

**Figure 3:**
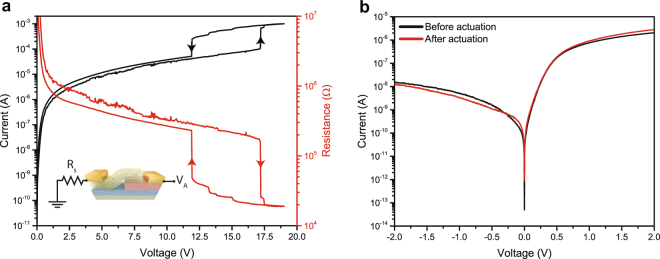
Electrically induced IMT of the VO_2_ side of the heterojunction. (**a**) Electrical characteristic of device D2 under large applied bias, sufficient to trigger the IMT of VO_2_ close to 17.5 V. Inset: biasing scheme used for the experiment. A discrete resistor R_S_ of 1 kΩ is connected in series to the device to limit the current in the low resistance state. (**b**) I–V curve of D2 measured before and after having electrically induced the IMT of VO_2_. The comparison between the two curves shows that the phase change of VO_2_ and the actuation power has no permanent effect on the electrical behavior of the heterojunction.

The capability of electrically inducing the IMT of the VO_2_ layer in the heterostructure in a room temperature experiment is shown in Fig. 3. This measurement has been performed on another device (D2) with the same structure of D1. The biasing configuration used for the measurements is the same shown in Fig. 1b, with the addition of a series resistance R_S_ of 1 kΩ to limit the maximum current. The I–V curve of the heterojunction (Fig. 3a) exhibits two steep jumps in correspondence of the IMT and MIT of VO_2_, separated by a hysteresis window. The voltage required to trigger the IMT is significantly larger with respect to the values that can be achieved in pure VO_2_ switches^4^ (see Supplementary Information Fig. S5). The high actuation voltage is due to the resistance of the heterojunction and to the relatively large distance between the MoS_2_ edge and VO_2_ metallic contact (more than 1 μm for D2). Remarkably, the proposed device delivers a lower leakage current in the diode subthreshold region with respect to pure VO_2_ switches. This feature combined with the possibility of modulating dynamically the IMT threshold by gating the MoS_2_ side of the junction could pave the way to more energy efficient VO_2_ switches. For the device of Fig. 3, the power thresholds required to electrically induce the IMT and MIT transitions from an n-n heterojunction to a Schottky diode and vice versa are respectively 1.83 mW and 2.89 mW. We verified that the actuation power density required to trigger the IMT did not alter the electrical behavior of the heterojunction by measuring the electrical characteristic of D2 before and after the electrical induced IMT. Figure 3b shows a direct comparison of the two I–V curves: no major variations are observed, demonstrating a full reversibility of the VO_2_ phase change and the stability of the heterojunction conduction.

### Photoresponse characterization

**Figure 4:**
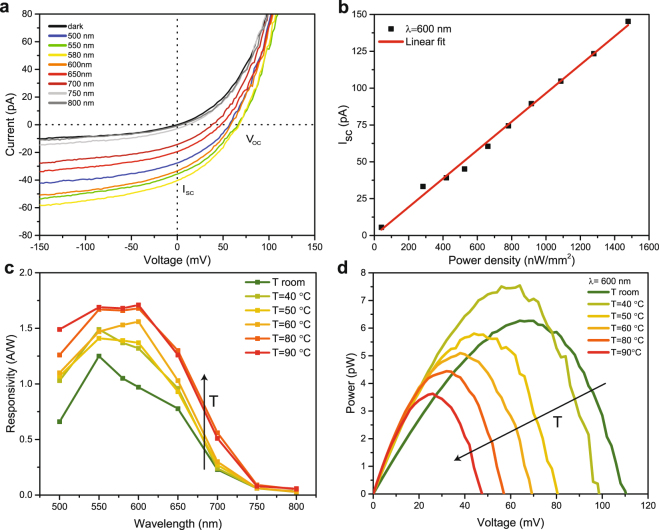
Photoresponse of MoS_2_/VO_2_ heterojunction. (**a**) I–V characteristic of D1 in linear scale under different illumination wavelengths, from 500 nm to 800 nm. The measurements have been performed under an incident power density close to 330 nW/mm^2^ for all the wavelengths and at room temperature. (**b**) Short circuit current of the heterostructure as a function of the incident power density, showing a linear dependency. (**c**) Spectral evolution of the photoresponsivity measured at different temperatures. At room temperature, a responsivity larger than the one of conventional silicon p-n photodiode is measured in the spectral range 500/650 nm. The responsivity in the visible spectrum is boosted by the increase of temperature and it saturates for temperatures above the IMT of VO_2_. (**d**) Electrical power generated by the photodiode under 600 nm illumination with an incident power density of 1.48 μW/mm^2^. The electrical power that the heterojunction can effectively harvest decreases with the increase of the temperature.

The photoresponse of device D1, whose dark I–V curve is reported in Fig. 2a, was characterized by illuminating the device with a focused light beam with controllable wavelength and power. The incident power density has been maintained well below the values required^3^ to optically trigger or affect VO_2_ IMT throughout all the measurements performed. The photosensitive area of the device corresponds to the overlap between the MoS_2_ flake and VO_2_ (see Fig. 1a), and it is estimated to be 104 µm^2^. Contributions to the photoresponse at zero applied bias from the MoS_2_ region far from the heterojunction have been found to be negligible coherently with the ohmic nature of the realized contacts (see Supplementary Information Fig. S1a,b). Moreover, the MoS_2_ region far from the junction shows a small contrast between the dark and illuminated IV curves measured across the set of contacts deposited on MoS_2_ (see Supplementary Information Fig. S1c). Figure 4a shows the impact of different illumination wavelengths with similar incident power density on the I–V curve of D1, resulting in a non-zero short circuit current I_SC_ and open circuit voltage V_OC_. A schematic representation of the origin of the photovoltaic effect in the heterojunction is reported in Supplementary Information Fig. S6. The measured I_SC_ shows a linear dependency with respect to the incident power density, as shown in Fig. 4b for illumination at 600 nm wavelength. We characterized the time domain photoresponse of the device by measuring the evolution of I_SC_ in response to ON/OFF and OFF/ON transitions of the light source. The device shows a symmetrical rise and decay time and the extracted response time at room temperature is 3.5 ms (see Supplementary Information Fig. S7). This value is considerably smaller than the ones reported in literature for several other implementations of MoS_2_ based photodetectors^20,21^ but still significantly larger than the response time in conventional Si photodiodes^21^.

We characterized the impact of temperature on the wavelength resolved photoresponsivity *R* at zero applied bias, defined as the ratio between the photogenerated current and the incident power on the device (Fig. 4c). The cut-off wavelength is located between 750 and 800 nm and it does not change with temperature, suggesting that light absorption happens mostly in the MoS_2_ side of the heterojunction^14^. At room temperature, a maximum photoresponsivity of 1.25 A/W was measured at 550 nm, and values exceeding silicon photodiode performance have been obtained in the 500/650 nm range^13^. Responsivity values larger than one have already been reported in p-n junctions based on doped multilayer MoS_2_ flakes, and can be attributed to the efficient photocarrier separation operated by the built in voltage of the junction^21,22^. The photoresponsivity in the visible range is clearly boosted by the temperature rise and tends to saturate above the VO_2_ IMT temperature. This enhancement can be explained by different mechanisms. First, the increase of VO_2_ work function with temperature^2,15^ determines a larger built-in voltage and therefore a stronger electric field at the junction that provides a more efficient separation of photogenerated carriers. Secondly, the MoS_2_ depleted thickness could increase because of VO_2_ work function and carrier density boost^18^, resulting in an increase of the photosensitive volume. Moreover, another contribution could come from the variation of the optical properties of VO_2_ producing potential optical interference effects responsible for an enhanced light absorption. Indeed, recent studies reported that the photoluminescence monolayer MoS_2_ flakes on VO_2_ substrates increases upon heating VO_2_ above the IMT temperature, most likely because of constructive optical interference^23,24^.

We extracted the generated electrical power P_el_ as a function of the applied voltage at different temperatures under 600 nm illumination with an incident power density of 1.48 μW/mm^2^ (Fig. 4d). The increase of temperature produces a boost of I_SC_ and a larger reverse current (Fig. 2c). This is related to a more favorable leakage path for photogenerated carriers, which causes a drop of V_OC_. Since V_OC_ decreases at a faster rate than I_SC_ grows (see Supplementary Information Fig. S8), the harvesting of the optical power becomes less efficient at high temperatures^14^. Therefore, the IMT of VO_2_ is beneficial for the heterostructure photosensitivity but appears detrimental for its photovoltaic performance.

## Discussion

In conclusion, we investigated a novel two-terminal device based on a VdW n-n heterojunction between VO_2_ and multilayer MoS_2_ flakes. The demonstrated devices show a rectifying electrical behavior, with rectification ratio larger than 10^3^, ideality factor of 1.75 and excellent forward conductance slope. The proposed heterojunction can be turned into a Schottky rectifier by electrically or thermally inducing the VO_2_ IMT. The electrically induced IMT is proved to be fully reversible and the MoS_2_/VO_2_ heterostructure grants a lower subthreshold leakage current with respect to pure VO_2_ switches. The envisioned possibility of modulating the IMT threshold voltage by gating the junction to electrostatically control the band alignment of the heterojunction could open a new category of VO_2_ based solid state devices with promising applications for steep slope electronic switches. Finally, we characterized the optical sensitivity of the heterojunction and obtained external photoresponsivities exceeding the results reported for several other implementations of photodiodes based on multilayer MoS_2_ flakes^11,13,14^. The fast transient response measured suggests that the device photoresponse is not dominated by trap-assisted optical absorption. The responsivity value is increasing with temperature and saturates above the VO_2_ IMT. The use of monolayer MoS_2_ for the realization of the heterostructure could result in a considerable enhancement of the photoresponsivity because of the direct band gap and the larger built-in voltage^23–25^. Large-area MoS_2_ synthesis techniques could allow a large scale, cheap fabrication of MoS_2_/VO_2_ heterojunction devices^26,27^. Moreover, recent reports of low-thermal budget deposition of VO_2_ thin films by pulsed laser deposition (PLD) are promising for the realization of CMOS compatible heterostructures^28^.

## Methods

### VO_2_ deposition

The 75 nm thick VO_2_ film was deposited on SiO_2_ substrate by reactive DC magnetron sputtering in high-vacuum conditions. The chamber was pumped to a base pressure lower than 5·10^-8^ mbar. The power on the V metal target (2 inch diameter, 99.95% purity) was set to 150 W. Ar process gas (purity 99.999%) was introduced in the chamber and the flow was regulated by a mass flow controller. During deposition, the oxygen pressure was kept constant by a Proportional Integral Derivative (PID) feedback control. This regulated the oxygen flow based on the pressure readings of a Zirox XS22 lambda-probe oxygen sensor. The process pressure was 7.45 ± 0.01·10^-3^ mbar and the oxygen partial pressure 4.86 ± 0.03·10^-4^ mbar. The temperature was measured by a stationary thermocouple above the rotating substrate holder and was kept constant at 600 °C. During deposition the substrate was rotating at 15 rpm. An *in situ* annealing was adopted during the slow cooling of the sample (30 °C/min).

### MoS_2_ synthesis

MoS_2_ powder was synthesized by heating a mixture containing stoichiometric amounts of molybdenum (99.9% pure, Alfa Aesar) and sulfur (99.999% pure, Alfa Aesar) at 1000 °C for 7 days in an evacuated and sealed quartz ampule. The mixture was slowly heated from room temperature to 1000 °C for 12 h in order to avoid any explosion due to the strong exothermic reaction and the high volatility of sulfur. From this powder, MoS_2_ crystals were grown using chemical vapor transport (CVT) with iodine as transport agent at ca. 5 mg/cm^3^. All quartz tubes used for vapor transport typically have an inner diameter of 16 mm and a length of 20 cm. The total powder charge is 5 g. A very slight excess of sulfur is always included (typically 0.5 wt % of the charge) to ensure the stoichiometry in the resulting crystals. The excess of sulfur is not incorporated into the dichalcogenide crystals but condenses as elemental sulfur onto the wall of the quartz tube at the end of the CVT process. The source and growth zones were kept at 1060 and 1010 °C, respectively, for 7 days in evacuated and sealed quartz ampules. After this time the furnace is turned off, a small fraction of the charge is transported toward the colder end of the tube, forming crystals with diameters of about 2−8 mm and thick tens of microns. The resulting crystals were washed with acetone and dried in vacuum. X-ray diffraction study has shown that MoS_2_ obtained in this way belongs to the 2 H polymorphism. A full characterization of the synthetized bulk samples is presented elsewhere^29^.

### Device fabrication

Electron beam lithography (EBL) on negative AZ nLof 2020:PGMEA 1:1 resist and wet etching in diluted commercial Cr etch solution were used to pattern the VO_2_ film sputtered on a 2 μm thick SiO_2_ layer. MoS_2_ multilayer flakes have been exfoliated on a PDMS stamp by using the scotch-tape micromechanical cleavage method and then deterministically transferred^30^ on the edge between VO_2_ pre-patterned structures and the SiO_2_ layer. The two-terminal devices were completed with a further EBL step and metal evaporation to deposit by lift-off 100 nm thick gold contacts on VO_2_ and MoS_2_. Gold was selected for the electrical connections in order to provide ohmic contacts to both VO_2_ and MoS_2_ (see Supplementary Information Fig. S1). The thickness of the transferred flakes was measured by atomic force microscopy, and the devices discussed in the text were realized with flakes thickness ranging from 80 to 100 nm.

### Electrical and optical characterization

All the measurements were carried on in ambient atmosphere and at temperatures ranging from ambient value to 100 °C. DC electrical measurements were performed using a HP4156A Semiconductor Parameter Analyzer and a Cascade Summit probe station. The light source used for optical characterization is a SuperK EXTREME supercontinuum white light lasers with a series SuperK SELECT multi-line tunable filter to select the wavelength within a minimum bandwidth of +/−5 nm. The optical power was measured with an 818-SL/DB Silicon Photodetector. The light beam was focused on the sample through optical microscopy lenses, resulting in a spot size of 0.25 mm^2^.

## Electronic supplementary material

Supplementary Information
