# Supplementary material for: Van der Waals MoS2/VO2 heterostructure junction with tunable rectifier behavior and efficient photoresponse
Source: Sci Rep. 2017 Oct 27;7:14250. doi: 10.1038/s41598-017-12950-y (PMC5660225; doi:10.1038/s41598-017-12950-y)
Supplement: Supplementary file 1 — Supplementary Information [file 41598_2017_12950_MOESM1_ESM.pdf]

Supplementary Information for

Van der Waals MoS2/VO2 heterostructure junction with tunable rectifier behavior and efficient photoresponse

Nicoló Oliva[1*], Emanuele Andrea Casu[1], Chen Yan[2], Anna Krammer[3], Teodor Rosca[1], Arnaud Magrez[4], Igor Stolichnov[1], Andreas Schueler[3], Olivier J.F. Martin[2] and Adrian Mihai Ionescu[1]

[1]Nanoelectronic Devices Laboratory (NanoLab), École Polytechnique Fédérale de Lausanne (EPFL), 1015 Lausanne, Switzerland

[2]Nanophotonics and Metrology Laboratory (NAM), École Polytechnique Fédérale de Lausanne (EPFL), 1015 Lausanne, Switzerland

[3]Solar Energy and Building Physics Laboratory (LESO-PB), École Polytechnique Fédérale de Lausanne (EPFL), 1015 Lausanne, Switzerland

[4]Istitut de Physique (IPHYS), École Polytechnique Fédérale de Lausanne (EPFL), 1015 Lausanne

1. OHMIC CONTACT TO MoS_2

We characterized the nature of the contact between MoS_2 flakes and gold electrodes by measuring the I-V characteristic between two set of contacts deposited on the same flake (see Fig. 1b). A good ohmic behavior is observed (Fig. S1a), and as shown in Fig. S1b there is no significant effect of light on the position of the I-V curve minimum. Fig. S1c shows a zoom of the I-V curve in linear scale in dark and under illumination. A very limited contrast between dark and illuminated current can be observed, since the MoS_2 cannot be electrostatically depleted. Therefore, the observed rectification behavior and photoresponse of the fabricated devices is fully deriving from the MoS_2/VO_2 heterojunction.

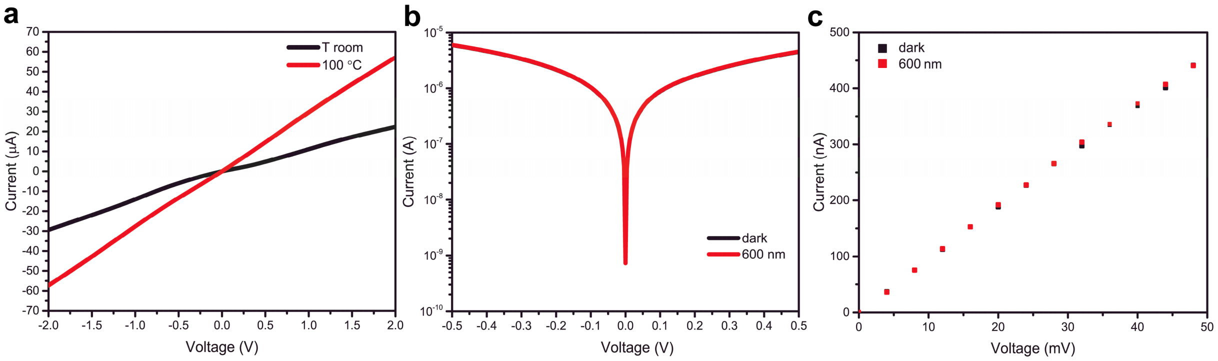

Figure S1. I-V curves of the electrical contacts to MoS_2 flakes a, Characteristic of the electrical contacts to MoS_2 at room temperature and 100 °C, showing a good ohmic behavior. **b,** Semi-logarithmic plot of the I-V curve at room temperature, under dark and illumination with 600 nm light with an incident power density of 1.48 $\mu W/mm^2$. There is no observable shift of the characteristic and the two curves are practically overlapped, demonstrating how the characterized photoresponse is coming only from the overlap region between MoS_2 and VO_2 in the proposed heterostructure. **c,** Zoom of the I-V curve in linear scale in dark and under illumination, showing a very limited contrast between the current under illumination and the current in dark condition.

2. XRD OF AS DEPOSITED VO_2 FILM AND RESISTIVITY CURVE

The XRD spectra of the deposited 75 nm thick VO_2 film is shown in Fig. S2a. A strong peak is clearly visible, perfectly corresponding to the (011) peak of monoclinic VO_2 phase, expected to be at 27.85° according to ICDD Powder Diffraction File Database - spectrum no 009-0142. Fig. S2b reports the resistivity curve of the VO_2 film measured in four probes configuration across the temperature induce metal to insulator transition. A resistivity reconfigurability of three orders of magnitude is observed, with the transition temperature close to 70 °C (340 K).[1]

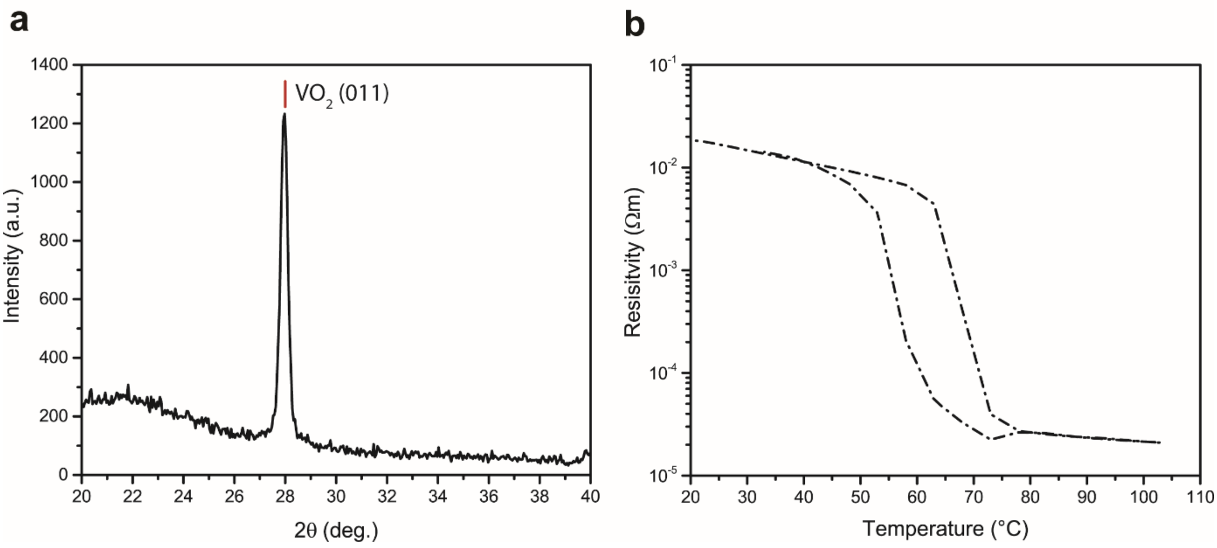

Figure S2. XRD characterization and resistivity curve of as deposited VO_2 film. a, XRD spectra and **b,** resistivity curve of as deposited 75 nm thick VO_2 thin films.

3. QUALITATIVE BAND DIAGRAM OF THE MoS_2/VO_2 HETEROSTRUCTURE

In Fig. S3 we show the band diagram drawn based on results reported in literature for both multilayer MoS_2 and VO_2. Although the workfunctions of the two materials have not been directly measured, the relevant parameters of the band diagram can be inferred based on established literature data. The procedure followed was:

- The conduction band discontinuity ΔE_C is estimated using: $\Delta E_C = \Phi_{VO_2} - X_{MoS_2}$ where Φ_{VO_2} is VO_2 workfunction while X_{MoS_2} is MoS_2 electron affinity.
- VO_2 workfunction, despite depending on film doping, is commonly reported in literature to be in the 4.8-5.2 eV range.[2-7]
- While bulk-like MoS_2 workfunction strongly depends on the chemical doping deriving from the synthesis procedure, its electron affinity is quite reliably reported to be close to 4 eV.[8-12]
- ΔE_C can be then estimated with a reduced margin of error to be in the 0.8-1.2 eV range. In the main text a value of 1 eV is considered in the discussion.
- The built-in voltage V_{bi} is then estimated to be in the 0.35-0.75 eV range.
- The Schottky barrier after the phase transition has been estimated using $\Phi_M = = \Phi_{VO_2} - X_{MoS_2} = 1.3$ eV, considering that VO_2 workfunction is reported to increase across the IMT of roughly 0.15 eV.[3]

The VdW gap present at the junction and the polycristallinity of the VO_2 film are not taken into account. The heterojunction present a type II band alignment when VO_2 is in the insulating state.

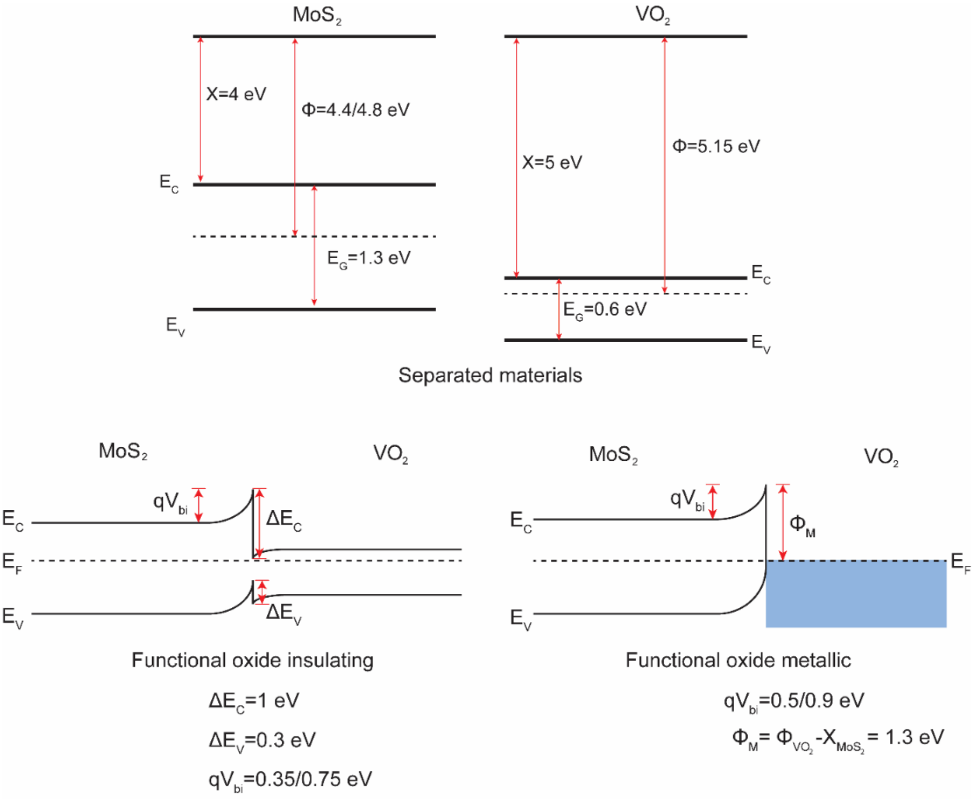

Figure S3. Band diagram of isolated MoS_2 and VO_2, and band alignment of the MoS_2/VO_2 heterojunction. Band diagram of the isolated materials and of the heterojunction.

4. JUNCTION CURRENT MODEL AND CURRENT SLOPE

The realized devices show a steep turn-on in the forward current as shown in Fig. 2a. In Fig. S4a we plot the extracted current slope $SS_I = (dlogI/dV)^{-1}$ of device D1 as a function of the junction current at room temperature. A current slope of 100 mV/dec is maintained over almost 4 orders of magnitude of the output current. However the extracted current slope in particular at low current values can be affected by the divergence of the I/V curve at 0 V,[13] so in the main test we extracted the conductance slope (Fig. 2d).

Fig. S4b shows the semi logarithmic I-V curve of D1 in the bias range +/- 0.3 V. In order to fit the experimental data we first used the Shockley diode equation:

$$I_D = I_S(e^{\frac{qV_a}{nk_BT}} - 1)$$

where I_S is the saturation current, V_a the applied voltage, n the diode ideality factor, and T the temperature. This expression of the device current provides a good fit of the forward current and allows to extract the diode ideality factor, but it fails to describe the non-saturating reverse current. In order to obtain a better fit of the reverse current we used the Fang and Howard model;[14] the saturation current expression is:

$$I_S = \frac{A_0}{T}(V_a + V_{bi})^{3/2} e^{\frac{-qV_{bi}}{k_BT}}$$

where A_0 is a parameter related to the heterostructure materials properties and V_{bi} is the built-in voltage at the junction. Imposing a built-in voltage of 0.6 V, compatible with the workfunction difference between VO_2 and MoS_2, we were able to obtain a good fit of both forward and reverse current, as shown in Fig. S4b.

We then extracted the ideality factor and the saturation current at different temperatures by fitting the forward current with the Shockley equation. The results are plotted in Fig. S4c. n increases across VO_2 IMT and then decreases for larger temperatures, with values between 1.75 and 2.1. The decrease of n above the IMT temperature has been observed as well for GaN/VO_2 heterostructures and it has been attributed to a reduction of interfacial states density once all the VO_2 grains become fully metallic.[2] The extracted values of the ideality factor suggest that the forward current of the heterojunction is characterized by two recombination mechanisms: minority carriers recombination in the neutral regions and recombination in the space charge region.[15]

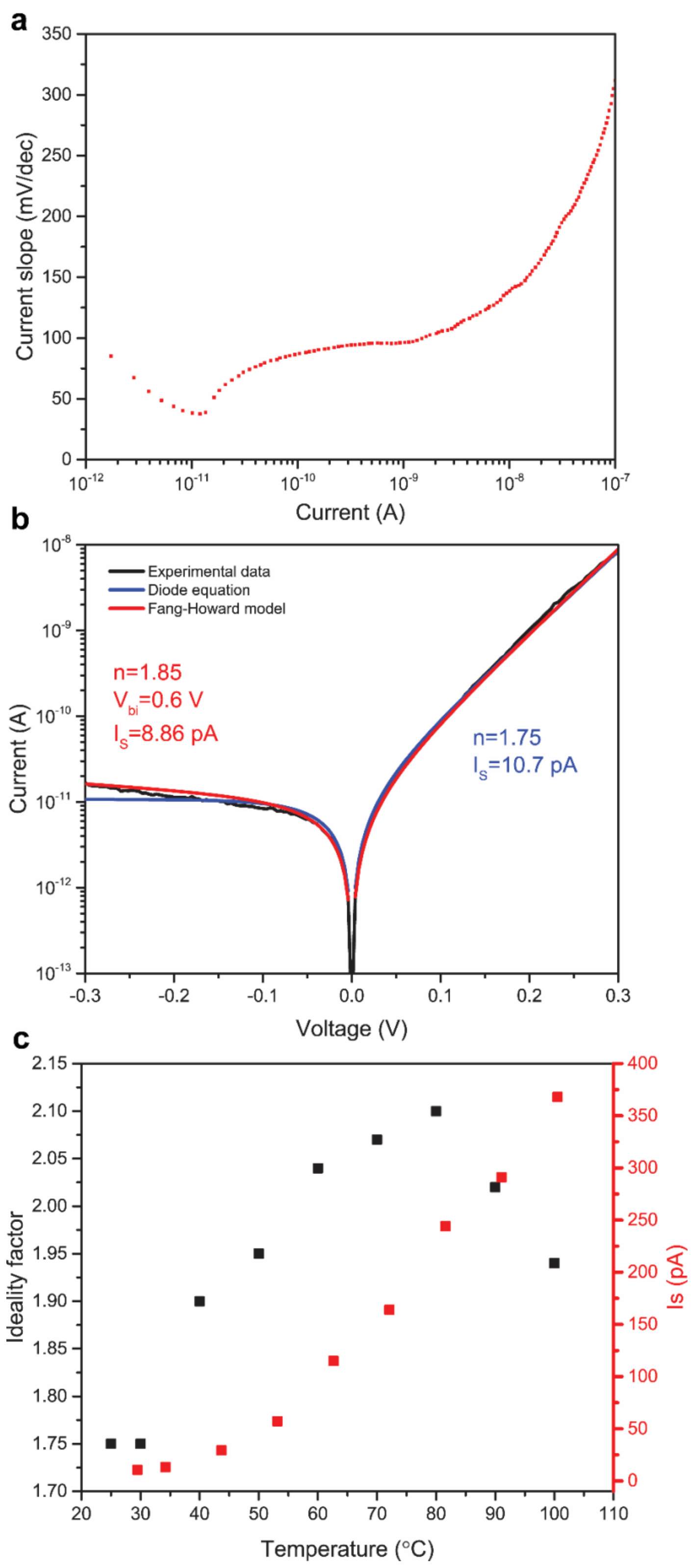

Figure S4. Current slope as a function of the junction current. a, Extracted current slope under forward bias for device D1. A good value of the slope around 100 mV/dec is maintained over 4 orders of magnitude of the output current. **b,** Fitting of the experimental I-V curve with the Shockley diode equation and the Fang-Howard model for the extraction of the saturation current and the diode ideality factor. c, Ideality factor at different temperatures. n increases across the IMT of VO_2 and then decreases for larger temperatures.

5. REFERENCE VO_2 SWITCH

In order to verify the quality of the sputtered VO_2 film we co-fabricated on the same substrate of devices D1 and D2 genuine VO_2 switches. Such devices are realized by depositing two gold contacts on the vanadium dioxide patterned structures. In Fig. S5 we plot the I-V and resistance curves of one of the switches measured at room temperature and with a series resistor of 1 kΩ. Comparing the VO_2 switch to the MoS_2/VO_2 heterostructure characteristic of Fig. 3a it is possible to notice that the threshold voltage for the electrically triggered IMT is much larger in the MoS_2/VO_2 heterojunction.

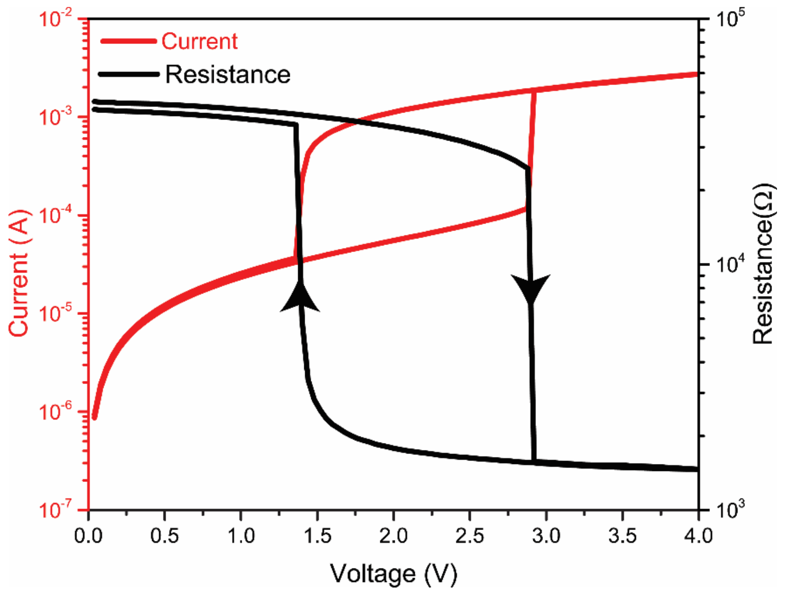

Figure S5. VO2 control switch electrically induced MIT. I-V characteristic of a pure VO_2 switch measured at room temperature connecting a 1 kΩ series resistor to limit the maximum current. The IMT is triggered close to 3V, and the reverse phase transition is separated by a hysteresis window of 1.5 V.

6. ORIGIN OF THE PHOTOVOLTAIC EFFECT AND STRENGTH OF THE ELECTRIC FIELD AT THE HETEROJUNCTION

The observed photovoltaic effect is due to the built-in voltage at the heterojunction that is responsible for the splitting of the photogenerated carrier pairs. Electrons are then collected at the contact on the MoS_2 side of the junction while holes are injected in VO_2. The band diagram in Fig. S6 provides a graphical representation of the origin of the photogenerated current. A similar mechanism has been reported for p-n junctions based on doped multilayer MoS_2 flakes, leading to high photoresponsivity values and relatively small response times.[16,17]

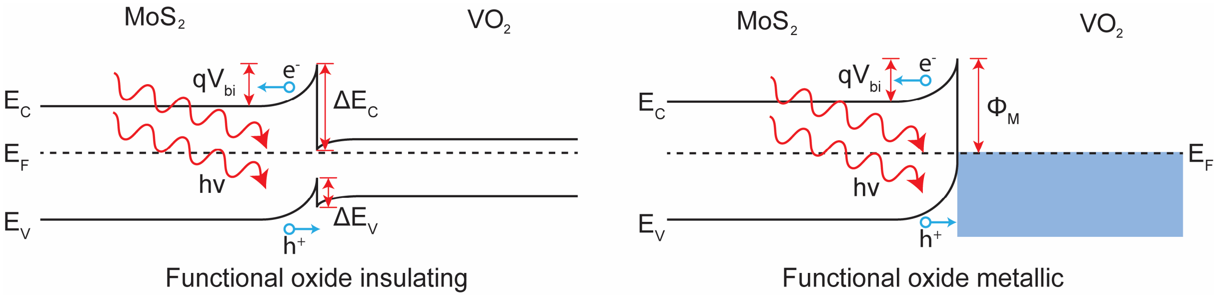

Figure S6. Band diagram of the heterojunction and origin of photocurrent with VO_2 in insulating and metallic state. The absorption of the incident light in the MoS_2 side of the junction determines the generation of an electron-hole pair. The photogenerated carriers are then split by the built-in electric field at the junction and collected at the MoS_2 (electrons) and VO_2 (holes) contact.

The built-in voltage in our devices is estimated to be between 0.35 eV and 0.75 eV. Most of the band bending takes place on the MoS_2 side because of the larger dielectric constant and electron concentration in VO_2.[2,12,18] The lowest limit for the electric field can be then estimated assuming that the MoS_2 flake is depleted through its entire thickness (maximum 100 nm). The obtained minimum value of the electric field at the junction is in the 35-75 kV/cm range.

7. TRANSIENT RESPONSE

In order to characterize the transient photoresponse of device D1, we measured in time domain the short circuit current transition obtained turning off the illumination source. The results are shown in Fig. S6, and have been obtained shining light at 600 nm with power density 1.48 µW/mm^2 for 35 ms and then turning off the light source. The measured decay time at room temperature is 3.5 ms, smaller than values reported in literature for other MoS_2 based photodetectors.[19-21] The reduced decay time suggests that the device photoresponse is not driven by long-lived trap states.

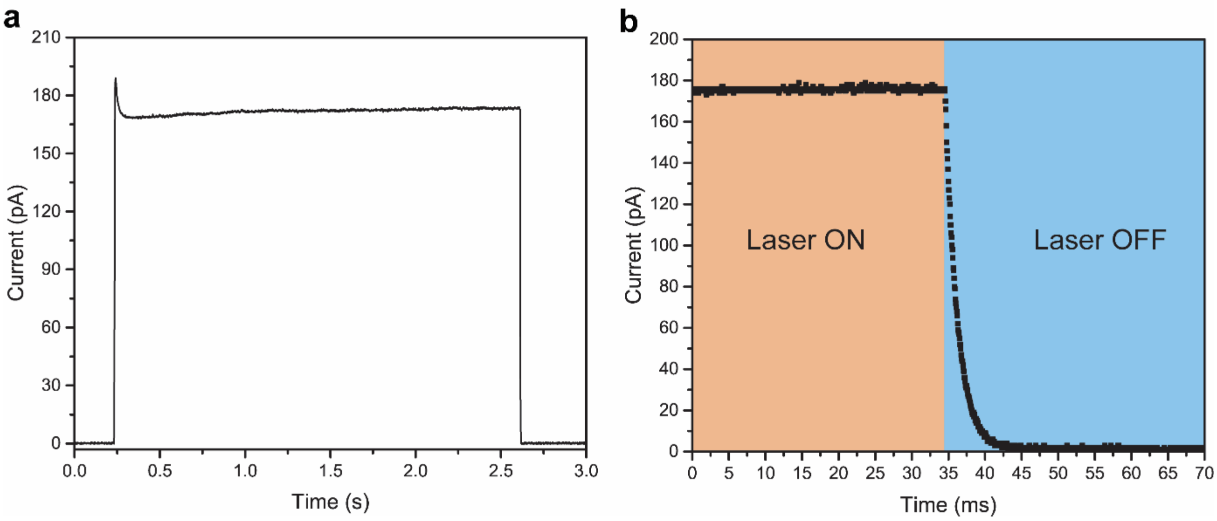

Figure S7. Transient photoresponse. a, Time domain photoresponse of D1 at zero applied bias and under illumination with 600 nm light at room temperature. The device shows a symmetrical rise and fall response. **b,** Transient response measured with an enhanced time resolution to estimate the decay time. The extracted decay time is 3.5 ms.

8. OPEN CIRCUIT VOLTAGE-SHORT CIRCUIT CURRENT VS TEMPERATURE

We report in Fig. S7 the measured open circuit voltage and short circuit current of device D1 under illumination with 600 nm light with power density 1.48 µW/mm^2 at increasing temperature. The maximum generated electrical power is directly proportional to the product of I_{SC} and V_{OC};[20] since the open circuit voltage decreases at a faster rate with respect to the short circuit current, the generated power decreases with the temperature as shown in Fig. 4d.

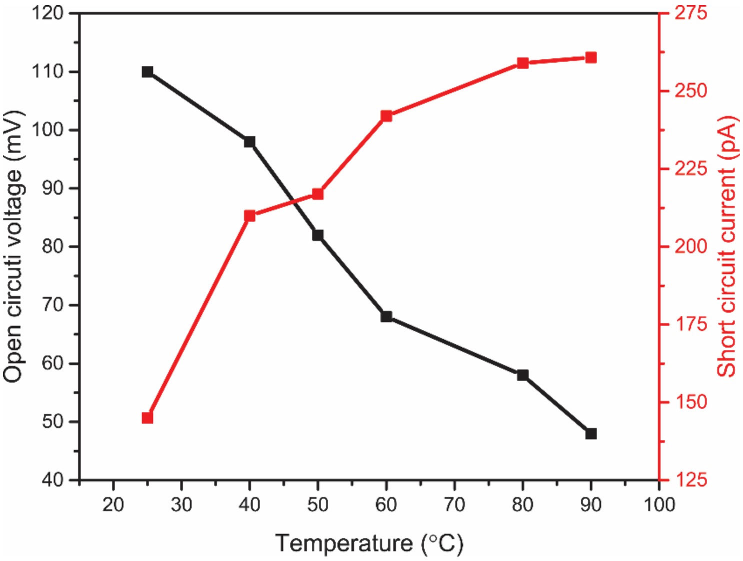

Figure S8. Measured V_{OC} and I_{SC} vs temperature. Open circuit voltage and short circuit current of device D1 measured under illumination with 600 nm light at 1.48 µW/mm^2 power density at different temperatures.

REFERENCES

1. Aetukuri, N. B. *et al.* Control of the metal–insulator transition in vanadium dioxide by modifying orbital occupancy. *Nat. Phys.* 9, 661–666 (2013).

2. Zhou, Y. & Ramanathan, S. GaN/VO2 heteroepitaxial p-n junctions: Band offset and minority carrier dynamics. *J. Appl. Phys.* **113,** (2013).

3. Ko, C., Yang, Z. & Ramanathan, S. Work function of vanadium dioxide thin films across the metal-insulator transition and the role of surface nonstoichiometry. *ACS Appl. Mater. Interfaces* **3,** 3396–401 (2011).

4. Sohn, A. *et al.* Evolution of local work function in epitaxial VO 2 thin films spanning the metal-insulator transition. *Appl. Phys. Lett.* **101,** 1–4 (2012).

5. Zhang, H.-T. *et al.* Wafer-scale growth of VO2 thin films using a combinatorial approach. *Nat. Commun.* **6,** 8475 (2015).

6. Sohn, A., Kanki, T., Sakai, K., Tanaka, H. & Kim, D.-W. Fractal Nature of Metallic and Insulating Domain Configurations in a VO2 Thin Film Revealed by Kelvin Probe Force Microscopy. *Sci. Rep.* **5,** 10417 (2015).

7. Martens, K. *et al.* The VO2 interface, the metal-insulator transition tunnel junction, and the metal-insulator transition switch On-Off resistance. *J. Appl. Phys.* **112,** (2012).

8. Li, Y., Xu, C.-Y., Wang, J.-Y. & Zhen, L. Photodiode-Like Behavior and Excellent Photoresponse of Vertical Si / Monolayer MoS2 Heterostrucutres. *Sci. Rep.* 1–8 (2014). doi:10.1038/srep07186

9. Tian, H. *et al.* Novel Field-Effect Schottky Barrier Transistors Based on Graphene-MoS2 Heterojunctions. 1–9 (2014). doi:10.1038/srep05951

10. Hao, L. *et al.* Electrical and photovoltaic characteristics of MoS2/Si p-n junctions. *J. Appl. Phys.* **117,** (2015).

11. Li, H.-M. *et al.* Metal-semiconductor barrier modulation for high photoresponse in transition metal dichalcogenide field effect transistors. *Sci. Rep.* **4,** 4041 (2014).

12. Das, S., Chen, H. Y., Penumatcha, A. V. & Appenzeller, J. High performance multilayer MoS2 transistors with scandium contacts. *Nano Lett.* **13,** 100–105 (2013).

13. Agarwal, S. & Yablonovitch, E. Band-Edge Steepness Obtained From Esaki/Backward Diode Current–Voltage Characteristics. *IEEE Trans. Electron Devices* **61,** 1488–1493 (2014).

14. Fang, F. F. & Howard, W. E. Effect of crystal orientation on Ge-GaAs heterojunctions. *J. Appl. Phys.* **35,** 612–617 (1964).

15. Shah, J. M., Li, Y. L., Gessmann, T. & Schubert, E. F. Experimental analysis and theoretical model for anomalously high ideality factors (n>>2.0) in AlGaN/GaN p-n junction diodes. *J. Appl. Phys.* **94,** 2627–2630 (2003).

16. Choi, M. S. *et al.* Lateral MoS2 p-n junction formed by chemical doping for use in high-performance optoelectronics. *ACS Nano* **8,** 9332–9340 (2014).

17. Zhong, X. *et al.* Multi-layered MoS 2 phototransistors as high performance photovoltaic cells and self-powered photodetectors. *RSC Adv.* **5,** 45239–45248 (2015).

18. Wi, S. *et al.* Enhancement of Photovoltaic Response in Multilayer MoS 2 Induced by Plasma Doping. *ACS Nano* 5270–5281 (2014). doi:10.1021/nn5013429

19. Lopez-Sanchez, O., Lembke, D., Kayci, M., Radenovic, A. & Kis, A. Ultrasensitive photodetectors based on monolayer MoS2. *Nat. Nanotechnol.* **8,** 497–501 (2013).

20. Svatek, S. A. *et al.* Gate Tunable Photovoltaic Effect in MoS_2 vertical P-N Homostructures. *J. Mater. Chem. C* **5,** 854–861 (2017).

21. Buscema, M. *et al.* Photocurrent generation with two-dimensional van der Waals semiconductors. *Chem Soc Rev* **44,** 3691–3718 (2015).
